# The Ethylene Signaling Pathway Negatively Impacts CBF/DREB-Regulated Cold Response in Soybean (*Glycine max*)

**DOI:** 10.3389/fpls.2019.00121

**Published:** 2019-02-12

**Authors:** Jennifer D. Robison, Yuji Yamasaki, Stephen K. Randall

**Affiliations:** Department of Biology, Indiana University–Purdue University Indianapolis, Indianapolis, IN, United States

**Keywords:** soybean, cold temperature, ethylene, proline, electrolytes, transcription factor, transcriptome, CBF/DREB

## Abstract

During cold stress, soybean CBF/DREB1 transcript levels increase rapidly; however, expected downstream targets appear unresponsive. Here, we asked whether the ethylene signaling pathway, which is enhanced in the cold can negatively regulate the soybean CBF/DREB1 cold responsive pathway; thus contributing to the relatively poor cold tolerance of soybean. Inhibition of the ethylene signaling pathway resulted in a significant increase in *GmDREB1A;1* and *GmDREB1A;2* transcripts, while stimulation led to decreased *GmDREB1A;1* and *GmDREB1B;1* transcripts. A cold responsive reporter construct (*AtRD29A_prom_::GFP/GUS*), as well as predicted downstream targets of soybean CBF/DREB1 [*Glyma.12g015100* (ADH), *Glyma.14g212200* (ubiquitin ligase), *Glyma.05g186700* (AP2), and *Glyma.19g014600* (CYP)] were impacted by the modulation of the ethylene signaling pathway. Photosynthetic parameters were affected by ethylene pathway stimulation, but only at control temperatures. Freezing tolerance (as measured by electrolyte leakage), free proline, and MDA; in both acclimated and non-acclimated plants were increased by silver nitrate but not by other ethylene pathway inhibitors. This work provides evidence that the ethylene signaling pathway, possibly through the action of EIN3, transcriptionally inhibits the CBF/DREB1 pathway in soybean.

## Introduction

Low temperature can be a limiting factor for plant growth and reproduction. Cold tolerant plants are able to modify gene expression resulting in higher survival rates during periods of low temperature (Gilmour et al., 1998; Thomashow, 1999). The most comprehensively studied cold responsive pathway is that regulated by the CBF/DREB1 (*C*-repeat responsive element *b*inding *f*actor/*d*ehydration-*r*esponsive *e*lement *b*inding factor) family of transcription factors (Liu et al., 1998; Thomashow, 1999; Park et al., 2015). These transcription factors contain an AP2-domain and bind the CRT/DRE (*C*-*r*epea*t*/*d*ehydration-*r*esponsive *e*lement) which has the core nucleotide sequence of CCGAC (Stockinger et al., 1997; Liu et al., 1998; Medina et al., 1999). Cold induction of CBF/DREB1 relies on the stabilization of the transcription factor ICE1 (*i*nducer of *C*BF *e*xpression 1) which activates CBF/DREB1 (Zarka et al., 2003). During normal temperatures, ICE1 is ubiquitinated by HOS1 (*h*igh expression of *os*motically responsive genes) and degraded by the 26S proteasome (Dong et al., 2006); however, in the cold ICE1 is sumoylated by the SUMO E3 ligase SIZ1 and phosphorylated by OST1 (*o*pen *st*omata 1) resulting in ICE1 stabilization and increased CBF/DREB1 expression (Miura et al., 2007; Ding et al., 2015). Cold stress induces significant upregulation of *AtCBF1, AtCBF2*, and *AtCBF3* (also known as *AtDREB1B, AtDREB1C, AtDREB1A*, respectively) within 15 min with maximal expression between 2 and 4 h (Gilmour et al., 1998). Downstream target transcripts containing CRT/DRE promoter elements significantly increase between 4 and 24 h later (Gilmour et al., 1998). Overexpression of *AtCBF1-3* in Arabidopsis leads to an increase of CRT/DRE containing transcripts and enhanced freezing survival (Jaglo-Ottosen et al., 1998). Conversely, when *CBF1-3* were knocked out via CRISPR/Cas9, Arabidopsis seedlings were hypersensitive to cold stress (Jia et al., 2016). *CBF/DREB1s* control 134 cold responsive genes (Jia et al., 2016), thus this regulon seems to be crucial to cold survival.

The exact mechanism by which the CBF regulon imparts cold tolerance remains incompletely characterized. However, metabolic changes resulting in the alteration of enzymes which combat oxidative damage, production of cryoprotective proteins, and changes in sugar content have all been reported (Steponkus et al., 1998; Cook et al., 2004; Gilmour et al., 2004; Kaplan et al., 2007; Hughes et al., 2013). Cold temperatures induce many physiological changes in plants, several of which are used to identify exposure or response to cold. During a period of cold temperatures, soluble sugars (Ristic and Ashworth, 1993) and free proline (Xin and Browse, 1998) accumulate in cold tolerant plants. Cold induces oxidative damage to lipids (O’Kane et al., 1996), which can be estimated by measuring the accumulation of malondialdehyde (MDA), an end product from the decomposition of lipid peroxidation (Janero, 1990). Photosynthesis is also disrupted by cold temperatures, exhibiting decreased electron transport rates, increased closed photosystem II (PSII) reaction centers, and decreased photosystem I (PSI) activity (Savitch et al., 2001).

Soybean (*Glycine max* [L.] Merr.) is an important agricultural species that is cold sensitive with severe tissue damage occurring near freezing temperatures and loss of vegetative growth below 6–7°C (Littlejohns and Tanner, 1976). Despite soybean’s cold sensitivity it does have the capability to cold acclimate, though its response is diminished related to cold-tolerant species (Robison et al., 2017). Within the soybean genome, 7 *CBF/DREB1* homologs have been identified: *GmDREB1A;1, GmDREB1A;2, GmDREB1B;1, GmDREB1B;2, GmDREB1C;1, GmDREB1D;1, GmDREB1D;2* (Kidokoro et al., 2015; Yamasaki and Randall, 2016). During cold stress, transcripts for all seven homologs were significantly upregulated after 1 h and remained elevated at 24 h (Yamasaki and Randall, 2016). However, predicted downstream CRT/DRE containing targets were largely unaffected by cold stress (Yamasaki et al., 2013; Yamasaki and Randall, 2016). When *GmDREB1A;1* and *GmDREB1A;2* were constitutively expressed in Arabidopsis the native CRT/DRE containing genes were upregulated in the absence of cold stress (Yamasaki and Randall, 2016), and enhanced freezing tolerance was imparted; indicating that *GmDREB1* transcription factors are capable of inducing CRT/DRE containing genes.

Ethylene is a versatile phytohormone that regulates a wide range of developmental and environmental responses (Street and Schaller, 2016). In the absence of ethylene, *c*onstitutive *t*riple *r*esponse (CTR1), phosphorylates *e*thylene-*in*sensitive 2 (EIN2) so that it remains inactive. However, when ethylene binds to the endoplasmic reticulum-membrane bound *et*hylene *r*eceptors (ETR1), CTR1 is deactivated. This results in proteolytic cleavage of EIN2, a serine/threonine Raf-like kinase, and translocation of the C-terminal fragment to the nucleus where it stabilizes *e*thylene-*in*sensitive 3 (EIN3) so that it is no longer degraded by *E*IN3 *b*inding *F*actor (EBF1/2) SCF ligases (Gallie, 2015; Ju and Chang, 2015). EIN3 is a transcription factor that binds to the consensus sequence ATGYATNY found in the promoters of ethylene responsive genes (Konishi and Yanagisawa, 2008; Boutrot et al., 2010). Ethylene regulation has a varied impact on cold stress across, and even within species. EIN3 can negatively impact cold tolerance, as *EIN3* over-expression mutants have decreased freezing tolerance while *ein3* knockouts have an increased freezing tolerance (Shi et al., 2012). Conversely, it has also been noted that the ethylene overproducer Arabidopsis mutant *eto1-3* has enhanced freezing tolerance (Catalá and Salinas, 2015). Ethylene production has also been linked to increased cold tolerance in grapevine (Sun et al., 2016) and tomato (Ciardi et al., 1997), while ethylene decreased cold tolerance in *Medicago truncatula* (Zhao et al., 2014), Bermuda grass (Hu et al., 2016), and tobacco (Zhang and Huang, 2010). The wide variety of roles ethylene plays in cold tolerance throughout the plant kingdom requires each species to be evaluated individually.

The perception or biosynthesis of ethylene can be chemically modulated. Stimulation of the ethylene signaling pathway is often accomplished using 1-aminocyclopropane-1-carboxylic acid (ACC) or ethephon (2-Chloroethylphosphonic acid). ACC is the biological precursor to ethylene in the biosynthetic pathway via the action of ACC oxidase, while ethephon is an ethylene producing molecule (Wang et al., 2002). Aminoethoxyvinylglycine (AVG), 1-methylcyclopropene (1-MCP), and silver ionic compounds are commonly utilized to inhibit the ethylene pathway. AVG inhibits ACC synthase, the rate limiting enzyme in the ethylene biosynthesis pathway, which produces ACC (Street and Schaller, 2016). 1-MCP is a competitive inhibitor for ethylene receptors, and silver ions are known to replace the copper ion within the ethylene receptor active site preventing activation even if ethylene is bound (Schaller and Binder, 2017).

The ethylene response in soybean has been well characterized at the reproductive stages. During early soybean reproduction (stage R1), inhibition of ethylene signaling with silver thiosulfate (STS), an ethylene perception inhibitor, resulted in a 55.6% increase in seed yield while ethylene production by application of ethephon, decreased seed yield by 50% and increased floral abscission rates (Cheng, 2013). Manipulation of ethylene homeostasis has several impacts on other hormonal pathways of reproductive age soybeans. Ethephon and STS treatments had opposite effects on the signaling pathways of auxin, abscisic acid, gibberellic acid, jasmonic acid, and salicylic acid (Cheng et al., 2013). Auxin, abscisic acid, and jasmonic acid signaling were increased with ethephon treatment, while gibberellic acid and salicylic acid signaling were stimulated by STS treatment (Cheng et al., 2013). Exposure to 1-MCP, an ethylene perception inhibitor, prior to heat stress resulted in increased chlorophyll content, photosynthetic efficiency, and decreased reactive oxygen species generation, and membrane damage compared to non-treated soybean (Djanaguiraman and Prasad, 2010). While much is known about ethylene signaling impacts in mature reproductive soybean, little information is available for the impact on younger soybean plants.

This study examines the role of ethylene regulation on the CBF/DREB1 cold stress pathway in soybean seedlings. First the physiological impact of ethylene signaling inhibitors in soybean seedlings was demonstrated and then the impact of manipulation of the ethylene pathway during cold treatment is examined.

## Materials and Methods

### Plant Materials and Growth Conditions

*Glycine max*, c.v Williams 82 was used in all experiments. Plants were grown in soil (BX Mycorrhizae^TM^, ProMix^®^) at 22°C under a long-day light cycle (16:8 light dark) with 180–200 μmol photon m^-1^ s^-1^. Initiation of treatments in all experiments took place 4 h after the lights came on (Zeitgeber+4) with 10–12 day old seedlings that had fully unrolled unifoliate leaves, stage VC (Hanway and Thompson, 1967). Unifoliate leaves were utilized in all experiments.

### Construction and Screening of Transgenic Plants Expressing RD29A_prom_::GFP/GUS

The Arabidopsis *RD29A* promoter (1,477 bp upstream of the start codon) was analyzed with plantCARE to identify stress responsive motifs present (Lescot et al., 2002). The *AtRD29A* promoter was amplified from Arabidopsis and cloned into pCambia1304 driving mGFP/GUS using Zero Blunt^®^ PCR Cloning Kit (ThermoFisher). The *AtRD29A_prom_::GFP/GUS* was ligated into pTF101.1 using EcoR1 and BamHI sites and then was introduced into *Agrobacterium* (strain EHA101) and used to transform half-seed explants cv Williams 82 using the bar gene for selection (Paz et al., 2004). The transformation and recovery of transgenic soybeans was performed by the Iowa State University Plant Transformation Facility^1^. Three independently transformed lines were selfed in a greenhouse to achieve homozygous lines, which were identified by herbicide resistance using 0.1% glufosinate (Finale^®^, Bayer) application to the midrib (Supplementary Figure S2).

### Soybean Ethylene Pathway Genes Homology and Expression

RNA-Seq data initially described in Yamasaki and Randall (2016) was examined for expression of ethylene pathway related genes. Briefly, in that experiment 10 day old soybean seedlings were exposed to 4°C for 0, 1, or 24 h prior to extraction of mRNA from unifoliate leaves. All treatments were performed in triplicate with *n* ≥ 6 plants per replication. Three libraries were created from the replicates for a total of nine libraries. Reads were mapped and statistically evaluated as described in Yamasaki and Randall (2016). All data can be found on NCBI GEO (Accession # GSE117686). A GO analysis (Ashburner et al., 2000; Consortium, 2017) was utilized to identify ethylene genes regulated by cold treatment. The predicted protein sequences of these genes were retrieved from Soybase ver. 2.1 (Grant et al., 2010) for soybean, and TAIR (Lamesch et al., 2012) for Arabidopsis. Clustal Omega (Sievers et al., 2011) was utilized to compare protein sequence homology. A phylogenetic tree to visualize the similarities of predicted protein sequences of Arabidopsis and soybean genes was generated and annotated with a heatmap by utilizing Interactive Tree of Life (Letunic and Bork, 2016) visualizing the log2 fold change of transcripts measured in the RNA-Seq analysis for soybean and microarray data from 4°C treated aerial portions of Arabidopsis (Kilian et al., 2007).

### Characterizing Ethylene Signaling Pathway Inhibition in Soybean Seedlings

**FIGURE 1:**
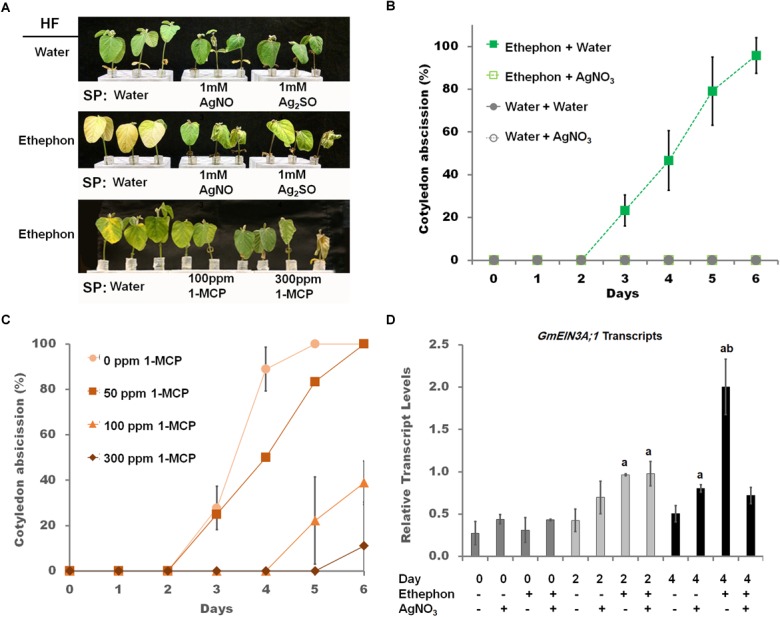
The effect of the ethylene receptor inhibitors, silver cation and 1-MCP, on soybean seedlings. **(A)** Effect of ethylene pathway inhibitor [1 mM silver nitrate, 1 mM silver sulfate, and 100 ppm 1-MCP foliar sprays (SP)] on cut seedlings hydroponically fed (HF) water for the first 24 h then either water or 1.38 mM ethephon for six additional days. Image is representative of three independent experiments. **(B)** Cotyledon abscission for seedlings sprayed (SP) with either water (closed symbols) or 1 mM silver nitrate (open symbols) 1 day prior to exposure to HF 1.38 ethephon (squares) or HF water (triangles). Foliar sprays continued daily over the entire monitoring period. Starting at 3 days, cotyledon abscission in seedlings HF ethephon and SP water was significantly increased based on Student’s *t*-test at the *p* < 0.01 level, which continued until the end of the monitoring period, *n* = 9. **(C)** Cotyledon abscission for seedlings sprayed with varying concentrations of 1-MCP starting 1 day prior to HF with 1.38 mM ethephon. Foliar sprays continued daily over the entire monitoring position, *n* = 9. **(D)** Transcript levels, determined by RT-qPCR, of *GmEIN3A;1* on days 0, 2, and 4 during 1 mM silver nitrate treatments from panel **(B)** and normalized to *GmACT11*. Two way ANOVA indicated significant impact of SP and HF along with an interaction between them at the *p* < 0.01 level (Supplementary Table S3). *Post-hoc* analysis (TukeyHSD) was used to compare samples, “a” indicates *p* < 0.01 comparing water (ethephon, AgNO_3_^-^) of the same day with the other treatments, *n* = 3 composed of a total of 12 plants. Error bars represent SD and those that are not visible are smaller than the symbol.

The impact of silver nitrate on ethylene responsiveness in soybean cotyledons was characterized using a cut seedling/hydroponic feeding method (Curtis, 1981). Only Figure 1 and Supplementary Figure S1 utilized this hydroponic method. Briefly, seedlings were cut 8–10 cm from the apical bud and placed in a 15 mL plastic Falcon tube which had been cut at the 5 mL line. Tubes were filled daily with either 1.38 mM ethephon (Sigma) or water as a mock control. Leaves were sprayed until runoff daily with either 1 mM silver nitrate (AgNO_3_, Fisher Scientific), 1 mM silver sulfate (Ag_2_SO^4^, Fisher Scientific), 50–300 ppm 1-Methylcyclopropene (1-MCP, AgroFresh) or water. Pre-treatment was accomplished by placing seedlings in tubes containing water and spraying with appropriate foliar sprays 24 h prior to being moved to new tubes containing either 1.38 mM ethephon or water. Cotyledon abscission was measured daily by counting the number of cotyledons that had fallen off after gentle agitation. Unifoliate leaves from individual plants were collected at various time points, frozen in liquid nitrogen, and stored at -80°C for later analysis.

### Ethylene Pathway Manipulation During Cold Stress

Wildtype Williams 82 and transgenic *AtRD29A_prom_::GFP/GUS* soybean lines (17-9, 22-23, and 28-5) were sprayed with 1 mM silver nitrate or mock control twice, 24 h before and immediately prior to transfer to 4°C. After 48 h in the cold, unifoliate leaves from 6 to 7 individual plants were collected for each replicate (18–21 total plants for three replicates for both mock and silver nitrate) and frozen in liquid nitrogen, then stored at -80°C for later analysis.

Transgenic line 22-23 seedlings of 14 days old were utilized for ethylene manipulation experiments. Seedling were sprayed with 1 mM silver nitrate, 100 ppm 1-MCP, 100 μM aminoethoxyvinylglycine (AVG, Sigma), 1 mM 1-Aminocyclopropane-1-carboxylic acid (ACC, Calbiochem), 1.38 mM ethephon or mock control at both 24 h and immediately prior to the start of cold treatment. Treatment concentrations for silver nitrate and 1-MCP were experimentally derived in this study, while AVG (Nukui et al., 2000), ACC (Zhang et al., 2010), and ethephon (Curtis, 1981) concentrations were obtained in the literature. Cold treatments were performed at 5°C for 48 h. The response of soybean to cold is for the plants to adopt a “sleep response” (unifoliate leaves folded down); Supplementary Figure S3. Time lapse photography (data not shown) showed this leaf movement response was initiated almost immediately following application of cold (only examined when cold was applied at *ZT* = 4) and was largely completed within 6 hours and was stable after that regardless of time of day or treatment until a return to control temperature. It should also be noted that silver treatment usually induced a red/purple coloration to the leaf veins and often resulted in leaf curling. The other treatments resulted in no significant differences compared to the cold control. Samples were flash-frozen in liquid nitrogen and then stored at -80°C until analysis.

### Chlorophyll Content

Chlorophyll was measured using a modified Warren (2008) microplate method. Leaves were pulverized in liquid nitrogen and 0.07 g was extracted with 0.7 mL 100% cold methanol. The tube was vortexed vigorously, covered in foil, and rotated for 5 min at room temperature prior to centrifugation at 10,000 rpm at 5°C. The supernatant was then collected and saved at -20°C, while the pellet was re-extracted with 0.7 mL and both supernatants were combined before 200 μL was added to a microplate and read on SpectraMax M2^®^ with PathCheck^TM^ using a methanol cuvette reference (Molecular Devices). Chlorophyll *a* and *b* were calculated using the equations in Warren (2008).

### Transcript Analysis

RNA was extracted via RNeasy^®^ Plant Mini Kit (Qiagen Cat. No. 74903) and treated with DNase (Qiagen Cat. No. 79254) during extraction. The cDNA was synthesized from 500 ng of RNA using SuperScript^®^ III First-Strand Synthesis using oligo(dT)_20_ primer (Invitrogen Cat. No. 18080051). Transcript levels were quantified via RT-qPCR starting with 6.25 ng cDNA, 500 nM of each primer (Supplementary Table S2) and 10 μL Maxima SYBR^®^ Green Master Mix (ThermoFisher) run on 7300 Real-Time PCR System (Applied Biosystems^®^) with a standard curve generated for each set of primers and normalized to either *GmACT11* or *GmUNK1* levels as described in Yamasaki and Randall (2016).

### GUS Assay

The GUS activity assay was modified from Yoo et al. (2007) and Fior et al. (2009). Total protein content was determined via Bradford Assay (Bradford, 1976). To assess GUS activity levels, 10 μg of total protein was combined with 100 μL MUG (4-Methylumbelliferyl-β-D-glucuronide dehydrate) substrate [10 mM Tris-HCl (pH 8), 1 mM MUG, and 2 mM MgCl_2_] in a black bottom 96 well plate. Fluorescence was measured every minute for 1 h at 37°C on a Spectramax M2^®^ (Molecular Devices) with excitation at 360 nm and emission at 460 nm. GUS activity was calculated from the linear slope of the fluorescence readings in R (R Core Team, 2013).

### Electrolyte Leakage

Six to eight replicates were tested for each variable with 3–4 leaf discs (1 cm diameter) randomly selected from all discs generated from 12 to 15 plants. Freezing was done in a glycerol bath (Brinkmann Lauda MGW RC 20) with temperatures starting at -1.0°C for 1 h at which time a single crystal of ice was added, and then lowered 0.5°C every 2 h until -4°C was reached then were maintained for another 2 h prior to overnight storage at 4°C. Three milliliters distilled water was added and vigorous shaking was applied for 6 h. Electrical conductivity was measured by a portable conductivity meter (Milwaukee Model MW301 EC meter). A subsequent freezing of the same samples at -80°C was used to determine 100% electrolyte leakage.

### Lipid Peroxidation and Proline Levels

Lipid peroxidation was measured using the 2-thiobarbituric acid-reactive substances (TBARS) assay which measures MDA concentration (Sharmin et al., 2012). Briefly, 50 mg of soybean leaf tissue was homogenized with a motor powered pestle in 0.5 mL of 20% trichloroacetic acid, 0.01% butylated hydroxytoluene, and 0.65% 2-thiobarbituiric acid. The samples were heated at 95°C for 30 min before being put on ice for 2 min. After centrifugation at 3 kg for 10 min, samples were read at 440, 453, and 600 nm on a SpectraMax M2^®^ microplate reader using PathCheck^TM^ with cuvette reference (Molecular Devices). MDA concentration was calculated using the equations of Hodges et al. (1999) to adjust for sucrose interference.

Proline was measured by the ninhydrin method (Bates et al., 1973). Briefly, leaf tissue (50 mg) previously collected, flash-frozen in liquid nitrogen, and stored at -80°C was pulverized and then extracted with 15 volumes of ethanol:water (4:6) overnight at 4°C. The ninhydrin reagent (200 μL) was heated to 95°C with 100 μL of extract for 20 min. Following cooling and a centrifugal spin to remove particulates, the absorbance of a 200 μL aliquot was measured in a microplate reader at 520 nm. A standard curve was generated from 0 to 30 nmoles proline.

### Photosynthetic Parameters

Chlorophyll *a* transient curves were measured using a Plant Efficiency Analyzer (Handy PEA, Hansatech). Fluorescence signal was recorded over 1 s of irradiation with an excitation light of 650 nm at 3,600 μmol m^-1^ s^-1^. Unifoliate leaves were dark adapted for 10 min with the clips provided with the Handy PEA. Clips were placed on the right side of the midrib approximately halfway between the leaf tip and base. There were nine unifoliate leaves from nine individual plants recorded for each condition. Parameters were analyzed using the JIP test (Strasser et al., 2000).

### Statistical Analysis

Two-way and one-way Analysis of Variance (ANOVA) with Tukey Honest Significant Difference *post-hoc* analysis were completed using R version 3.3.1 (R Core Team, 2013). Two-tailed Student’s *t*-test was calculated in Excel (Microsoft, 2013).

## Results

### Ethylene Responsiveness and Ethylene Pathway Inhibition in Soybean Seedlings

To measure ethylene responsiveness in soybean seedlings, several targets of ethylene, leaf senescence (Grbić and Bleecker, 1995), cotyledon abscission (Curtis, 1981), and chlorophyll degradation (Purvis and Barmore, 1981), were examined. In excised seedlings no abscission of the cotyledons occurred (Figure 1A, top row, Figure 1B). Hydroponic feeding (HF) of 1.38 mM ethephon, a bioconvertible precursor to ethylene (Murray et al., Hort sci, 1995), resulted in full cotyledon abscission and increased leaf yellowing. The concentration of ethephon used was the same as Curtis (1981). When ethylene binding inhibitors (1-MCP, silver nitrate, and silver sulfate) were applied as foliar sprays (SP), both abscission and yellowing were diminished (Figure 1A, bottom two rows, Figure 1B). Seedlings sprayed with silver nitrate simultaneously with ethephon hydroponic feeding lead to a delay of abscission, inhibition of chlorophyll *a* loss, and a delayed reduction of *GmEIN3A;1* transcript levels (Supplementary Figures S1A–C). However, when silver nitrate treatment preceded ethephon by 1 day, cotyledon abscission was completely blocked (Figure 1B). Complete cotyledon abscission blockage was only achieved with 1 mM silver nitrate, only partial prevention of abscission was noted with 0.125, 0.5, or 0.175 mM silver nitrate (data not shown). To optimize foliar spray of 1-MCP concentrations, 50, 100, and 150 ppm were applied to ethephon hydroponically fed seedlings. Increasing concentrations of 1-MCP also increased the delay of cotyledon abscission (Figure 1C), 100 ppm was identified as the optimal concentration of 1-MCP (Figure 1A bottom row). At 4 days of treatments, when approximately 50% of cotyledons had abscissed in the ethephon treated plants; a marked impact on *GmEIN3A;1* transcript was observed. *GmEIN3A;1* transcripts were approximately four times that of the non-treated leaves and silver treatment reduced *GmEIN3A;1* levels to near control levels (1.4×, Figure 1D).

### Changes in Ethylene Pathway Transcripts in Cold-Treated Soybeans

**FIGURE 2:**
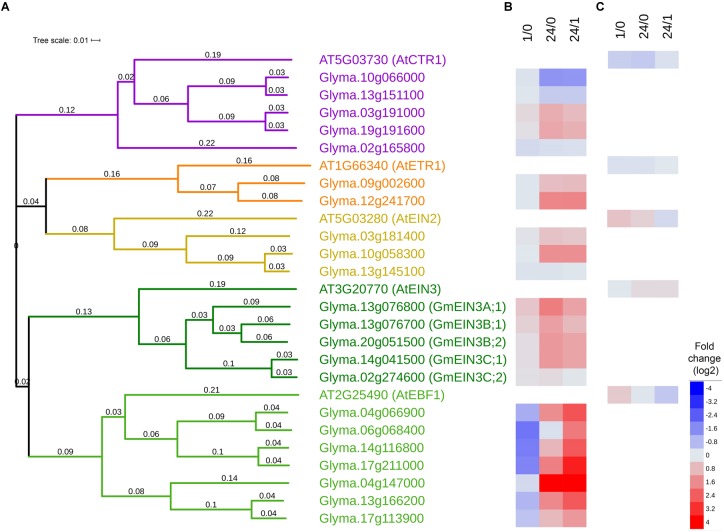
Ethylene signaling pathway genes identified in RNA-Seq from cold treated soybean. **(A)** Phylogeny tree depicting predicted protein sequences from Arabidopsis [extracted from TAIR (Lamesch et al., 2012)] and soybean [extracted from Soybase (Grant et al., 2010; Severin et al., 2010)] homologs. The colorations on the tree reflect the protein classification with purple indicating serine/threonine-protein kinase, yellow/orange indicating membrane bound, and green indicating transcription factors. **(B)** Heatmap of log2 fold changes comparing soybean cold responses (4°C) 1 vs 0 h (1/0), 24 vs 0 h (24/0), and 24 vs 1 h (24/1) from RNA-Seq data (from Yamasaki and Randall, 2016) where blue indicates decreases and red indicates increases in comparison. The averaged read counts with SDs are in Supplementary Table S1. **(C)** Heatmap of log2 fold changes comparing cold responses (4°C) of the aerial portions of Arabidopsis 1 vs 0 h (1/0), 24 vs 0 h (24/0), and 24 vs 1 h (24/1) from microarray data from Kilian et al. (2007) where blue indicates decreases and red indicates increases in comparison.

Several key ethylene signaling transcripts were significantly upregulated by cold (Figure 2 and Supplementary Table S1). Five predicted homologs of *AtCTR* were identified in soybean, three of which (*Glyma.10g066000, Glyma.13g151100, Glyma.02g165800*) were downregulated and two (*Glyma.03g191000* and *Glyma.19g1916000*) were upregulated by 24 h of cold exposure. Two predicted homologs of the ethylene receptor *AtETR1* (*Glyma.09g002600* and *Glyma.12g241700*) were upregulated by 24 h of cold exposure. Of the three predicted *EIN2* homologs, two (*Glyma.03g181400, Glyma.10g058300*) were significantly increased at 24 h, where one (*Glyma13g20810*) remained unchanged. Of five predicted *EIN3* homologs, four (*Glyma.13g076800, Glyma.13g076700, Glyma.20g051500, Glyma.14g041500*) were accumulated greater than twofold after 24 h in the cold. The genes that increased in the cold were designated *GmEIN3A;1, GmEIN3B;1, GmEIN3B;2* and *GmEIN3C;1* while the one that did not increase Glyma.02g27460 was designated *GmEIN3C;2*. The significant increase of *GmEIN3* transcripts in cold treated soybean seedlings after 24 h (ranged from 2.5 to 3 fold in the three most responsive *GmEIN3* genes) is distinct from the modest effect in aerial portions of Arabidopsis (log2 0.2 or 1.2 fold; Kilian et al., 2007) or approximately 1.3 fold at 24 h in whole Arabidopsis seedlings (Shi et al., 2012). Additionally, of the seven predicted EBF1 homologs, four (*Glyma.04g066900, Glyma.06g068400, Glyma.14g116800, Glyma.17g211000*) were significantly decreased after 1 h of cold stress. After 24 h of cold exposure, six soybean *EBF* homologs were upregulated. The immediate activation of the ethylene signaling pathway in the cold is demonstrated by the upregulation of positive regulators (*EIN2* and *EIN3*) and the concomitant transient decrease of negative regulators (*EBF1*) of the pathway.

### Manipulation of the Ethylene Signaling Pathway During Cold Stress

**FIGURE 3:**
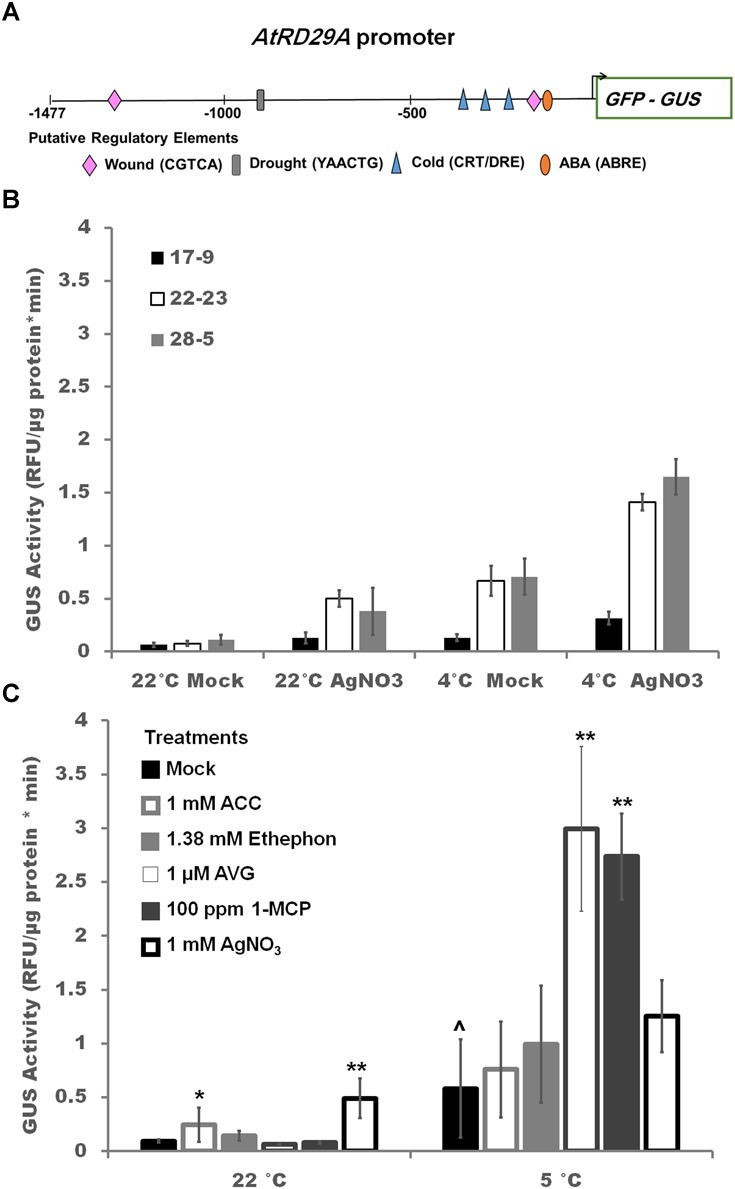
Reporter response to ethylene pathway modulations **(A)** The *AtRD29A_prom_::GFP/GUS* construct introduced into soybean with the major abiotic stress regulatory elements highlighted. **(B)** Effect of 1 mM silver nitrate treatment sprayed twice (–1 and 0 day) prior to exposure to cold or control temperatures for 2 days in three independent homozygous transgenic soybean lines. Two Way ANOVA showed an independent effect of temperature and treatment at *p* < 0.001 and combinatorial effect of treatment^∗^temperature of *p* < 0.01 (Supplementary Table S3). **(C)** Effect of ethylene pathway stimulators (1 mM ACC, 1.38 mM ethephon) and inhibitors (1 μM AVG, 100 ppm 1-MCP, 1 mM AgNO_3_) on GUS activity in transgenic soybean line ST164-22-23. One way ANOVA was performed for each temperature individually (Supplementary Table S3). *Post-hoc* analysis using TukeyHSD was performed comparing treatment with the mock from the same temperature, ^∗^*p* < 0.05, ^∗∗^*p* < 0.01. Comparison between cold treated and non-cold treated mocks was done using Student’s *t*-test, ^∧^*p* < 0.05. *n* = 3 composed of a total of 12 plants.

The abiotic stress reporter contains the GFP/GUS fusion protein driven by the native *AtRD29A* promoter which possesses three CRT/DRE elements, the binding site for CBF/DREB1, two abscisic acid responsive element (ABRE), one drought responsive MYB binding site (MBS) element, and two methyl-jasmonate wound responsive (CGTCA) motifs (Figure 3A; Sazegari et al., 2015). GUS activity varied approximately twofold among the three independent homozygous lines in the absence of stress (Figure 3B). GUS activity level increased in all three soybean transgenic lines during cold stress by fold 2.1, 6.4, 8.7 (line 17-9, 28-5, 22-23, respectively) after 2 days (Figure 3B). Pre-treatment with silver nitrate resulted in a 2.0, 3.5, 6.5 fold increase at 22°C indicating that silver nitrate alone is sufficient to upregulate the *AtRD29A* driven construct (Figure 3B). The addition of cold-treatment (4°C) to silver treated seedlings resulted in an additional increase (2.3, 2.4, 2.1 fold) in all lines (Figure 3B). To address specificity for the observed ethylene regulation of *AtRD29A_prom_::GFP/GUS* expression, line 22-23 was further subjected to modulators of different specificity; inhibitors (1 μM AVG, 100 ppm 1-MCP) and stimulators (1.38 mM ethephon, 1 mM ACC) during cold stress (Figure 3C). Compared to cold stressed, mock treated soybean, treatment with ethylene inhibitors (AVG, 1-MCP, though not silver nitrate) significantly increased GUS activity (Figure 3C). This supports the hypothesis that the increase in GUS activity is due to release of ethylene inhibition of the cold response.

**FIGURE 4:**
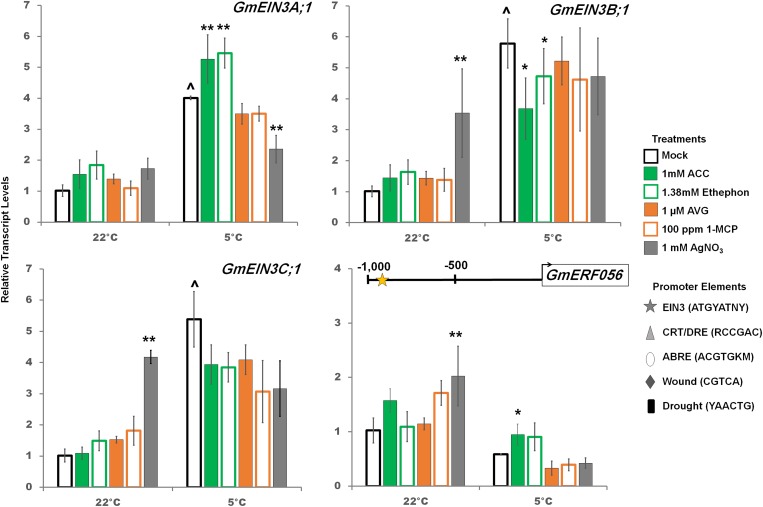
RT-qPCR analysis of transcript levels of *GmEIN3A;1, GmEIN3B;1* and *GmEIN3C;1*; as well as a predicted downstream target of EIN3, *GmERF056*, with ethylene pathway stimulation (1 mM ACC, 1.38 mM ethephon) or inhibition (1 μM AVG, 100 ppm 1-MCP, 1 mM AgNO_3_) after 2 days at either control or cold temperatures. Transcripts normalized to *GmUNK1* transcript levels. Position of the consensus EIN3 binding elements present in the promoter of *GmERF056* is displayed above the graph. All promoters depicted in Figure 4–6 were examined for the consensus binding elements shown. Error bars represent SD. One way ANOVA was performed for each temperature individually (Supplementary Table S3). *Post-hoc* analysis using TukeyHSD was performed comparing treatment with the mock from the same temperature, ^∗^*p* < 0.05, ^∗∗^*p* < 0.01. Comparison between cold treated and non-cold treated mocks was done using Student’s *t*-test, ^∧^*p* < 0.05. *n* = 3 composed of a total of 18–21 plants.

RNA-Seq analysis of soybean *GmEIN3s* indicated differential regulation of the various *GmEIN3* transcripts in the cold (Figure 2). Only *GmEIN3A;1, GmEIN3B;1, GmEIN3B;2* and *GmEIN3C;1* were cold induced and within those *GmEIN3A;1* had the highest cold induction of 3.6 fold after 24 h (Supplementary Table S1). Three cold inducible *GmEIN3s* were validated by RT-qPCR (Figure 4). In the cold, *GmEIN3A;1* transcripts were increased by ethylene pathway stimulation and trended downward with ethylene pathway inhibition, though only silver nitrate treatment was significantly decreased (Figure 4). Conversely, *GmEIN3B;1* transcript levels were decreased by ethylene pathway stimulation in the cold, while *GmEIN3C;1* was unaffected by any ethylene pathway manipulation (Figure 4). A downstream target of EIN3, E*RF056* (*Glyma.15g15200*) which possesses an consensus EIN3 binding site at -976 bp, was not cold induced, but was increased significantly with ACC treatment (Figure 4).

**FIGURE 5:**
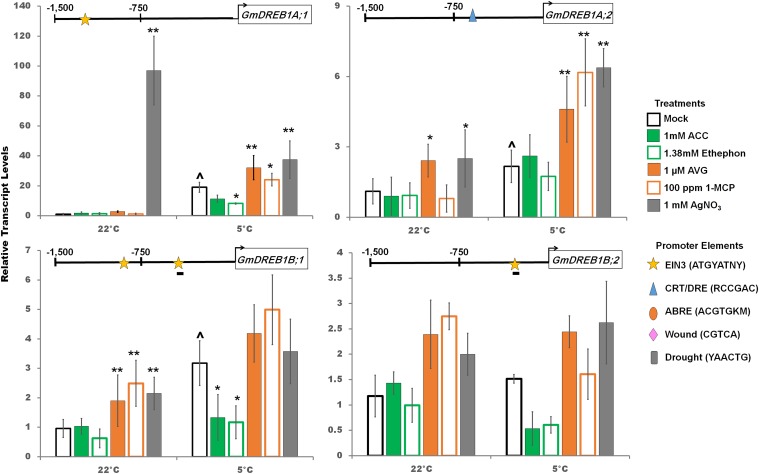
Changes in transcript levels (RT-qPCR) of *GmDREB1A;1, GmDREB1A;2, GmDREB1B;1* and *GmDREB1B;2* by ethylene pathway stimulators (1 mM ACC, 1.38 mM ethephon) or inhibitors(1 μM AVG, 100 ppm 1-MCP, 1 mM AgNO_3_) after 2 days at either control or cold temperatures. Transcripts normalized to *GmUNK1* transcript levels. All promoters depicted in Figure 4–6 were examined for the consensus binding elements shown. Binding elements present in promoters displayed above the graph of each transcript. A line beneath the motif symbol indicates the motif is found on the reverse strand. Error bars represent SD. One way ANOVA was performed for each temperature individually (Supplementary Table S3). *Post-hoc* analysis using TukeyHSD was performed comparing treatment with the mock from the same temperature, ^∗^*p* < 0.05, ^∗∗^*p* < 0.01. Comparison between cold treated and non-cold treated mocks was done using Student’s *t*-test, ^∧^*p* < 0.05. *n* = 3 composed of a total of 18–21 plants.

Cold responsive *GmCBF/DREB1* transcripts are strongly, but transiently, accumulated in response to cold, generally declining after 10 h of cold onset, and reaching a more constant but still increased level, compared to non-cold treated plants, after several days (Yamasaki and Randall, 2016). Despite this rapid reaction, soybean does not attain significant cold tolerance until after 2 days of cold and approaches a maximum acclimation after 7 days (Robison et al., 2017). Therefore, it was decided to examine the various molecular and physiological responses at 2 days post cold treatment, especially since at least 3 days (starting with 1 day before onset of cold treatment) of silver nitrate treatment was required to impact cotyledon abscission and decreased *GmEIN3A;1* transcripts (Figure 1B,D and Supplementary Figure S1C). Transcript levels of the *GmCBF/DREB1* family were measured 2 days after cold with stimulation or inhibition of the ethylene signaling pathway (Figure 5). *GmDREB1A;1, GmDREB1A;2* and *GmDREB1B;1* were significantly cold induced after 2 days in the cold while *GmDREB1B;2* was not. *GmDREB1A:1* had the greatest (sustained) fold-increase after 2 days in the cold, as well as the most abundant transcript at 0, 1, and 24 h based on RNA-Seq (Supplementary Table S1) and total transcript copy number based on RT-qPCR (Yamasaki and Randall, 2016). The promoters of *GmDREB1A;1, GmDREB1B;1* and *GmDREB1B;2* had predicted EIN3 promoter elements (yellow stars, Figure 5). In the cold, ethylene pathway stimulation decreased *GmDREB1A;1* transcript levels, while ethylene pathway inhibition increased transcript levels (Figure 5). In the cold, *GmDREB1A;2* transcripts were increased with ethylene pathway inhibition despite the lack of obvious EIN3 binding sequences in the promoter (Figure 5). However, *GmDREB1A;2* does contain a predicted CRT/DRE promoter element (blue triangle) and may be responding to the increasing *GmDREB1A;1* transcript levels (Figure 5). In the cold, *GmDREB1B;1* and *GmDREB1B;2* tended to decrease with ethylene pathway stimulation and increase with ethylene pathway inhibition; however, not always statistically significant at the 0.05% level (Figure 5).

**FIGURE 6:**
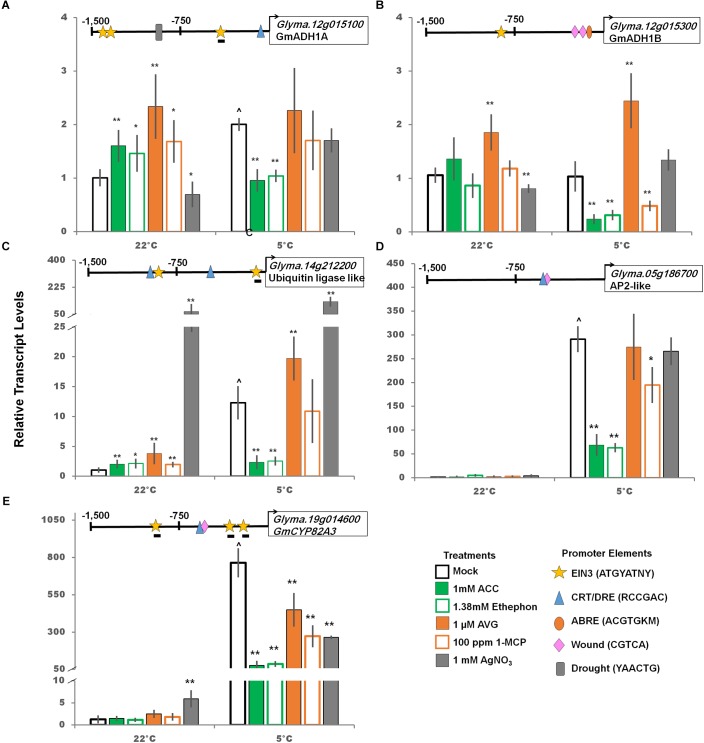
Impact of ethylene pathway stimulation and inhibition on transcript levels of cold responsive genes (panels **A-E**, *GmADH1A, GmADH1B*, ubiquitin ligase-like, AP2-like, and *GmCYP82A3*; respectively) after 2 days at either control or cold temperatures measured via qRT-PCR and normalized to *GmUNK1*. All promoters depicted in Figure 4–6 were examined for the consensus transcription factor binding elements shown. Binding elements present in promoters displayed above the graph of each transcript. A line beneath the motif symbol indicate the motif is found on the reverse strand. Error bars represent SD. One way ANOVA was performed for each temperature individually (Supplementary Table S3). *Post-hoc* analysis using TukeyHSD was performed comparing treatment with the mock from the same temperature, ^∗^*p* < 0.05, ^∗∗^*p* < 0.01. Comparison between cold treated and non-cold treated mocks was done using Student’s *t*-test, ^∧^*p* < 0.05. *n* = 3 composed of a total of 18–21 plants.

Predicted downstream targets of *GmCBF/DREB1* were examined. Two alcohol dehydrogenase (ADH1) like soybean genes, one containing a predicted CRT/DRE promoter element (*Glyma.12g015100*) and one without (*Glyma.12g015300*) were compared as ADH is thought to play an important role in cold and freezing tolerance (Song et al., 2016). *Glyma.12g015100* transcripts were cold induced, as expected due to presence of CRT/DRE in the promoter, and treatment with ethylene pathway stimulators decreased transcript levels to non-cold treated levels. However, there was no increase when treated with ethylene pathway inhibitors (Figure 6). Though not cold-induced *Glyma.12g015300* was impacted by ethylene pathway manipulation, possibly due to interactions at one or more of two drought, one ABRE, and one EIN3 binding motifs predicted.

Three additional potential targets of the CBF/DREB1s, annotated as a ubiquitin ligase (Glyma.14g212200), an AP2-like transcription factor (*Glyma.05g186700*), and *GmCYP82A3* *(Glyma.19g041600*), a cytochrome P450 (Yan et al., 2016), were strongly accumulated in response to the cold (Figure 6 and Supplementary Table S1). Furthermore, transcript levels of all three significantly decreased when treated with ethylene stimulator treatment in the cold (Figure 6). Interestingly, *GmCYP82A3* transcript levels decreased with both ethylene stimulation and inhibition compared to the cold control. The promoter for this gene, in addition to the CRT/DRE, also includes three EIN3 binding elements and a wound response element, suggesting this complex response could be due to crosstalk between these pathways (Figure 6).

### Impact of Ethylene Pathway Modulators on Freezing Tolerance, Oxidation, Proline Levels, and Photosynthesis

**FIGURE 7:**
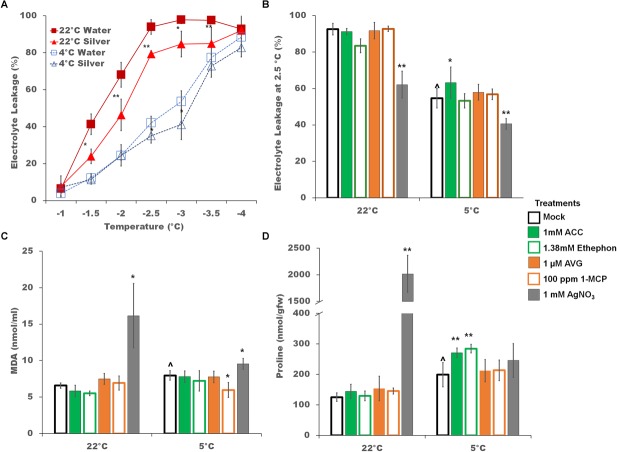
Physiological responses to ethylene pathway manipulation during cold stress. **(A)** Freezing tolerance of soybean seedlings treated with silver nitrate (triangles) or mock controls (squares) as measured by electrolyte leakage across a range of freezing temperatures after 2 days of either control (22°C, red lines) or cold (5°C, blue lines) temperatures. **(B)** Freezing tolerance at –2.5°C of soybean seedlings during ethylene pathway stimulation or inhibition. **(C)** MDA levels of soybean seedlings during ethylene pathway stimulation or inhibition. **(D)** Free proline levels of soybean seedlings during ethylene pathway stimulation or inhibition. Error bars represent SD and non-visible error bars are smaller than the symbol. One way ANOVA was performed for each temperature individually (Supplementary Table S3). *Post-hoc* analysis using TukeyHSD was performed comparing treatment with the mock from the same temperature, ^∗^*p* < 0.05, ^∗∗^*p* < 0.01. Comparison between cold treated and non-cold treated mocks was done using Student’s *t*-test, ^∧^*p* < 0.05. *n* = 3 composed of a total of 18–21 plants.

The finding that the *GmDREB1A;1* and *GmDREB1A;2* transcripts increased in the presence of silver nitrate during cold treatment (Figure 4); suggested that silver nitrate treatment might enhance cold tolerance. After 2 days, non-acclimated silver nitrate treated plants had significantly better freezing tolerance between -1.5 and -3.5°C; and cold acclimated plants demonstrated a lesser though significant, impact. The LT50 for non-acclimated plants was -1.7 and -1.9°C (with and without silver nitrate, respectively) and -2.7 and -2.9°C for cold acclimated plants (Figure 7A). Electrolyte leakage at -2.5°C was tested for all ethylene pathway stimulators and inhibitors, and their impact was found to be minimal.

Oxidation of membrane lipids is a common environmental damage, typically assessed by measuring changes in MDA levels (Hodges et al., 1999). In soybean seedlings, MDA content was cold induced (Figure 7C). Overall, ethylene signaling pathway manipulation had no strong additional effect on lipid oxidation in cold treated plants (Figure 7C). At 22°C, silver nitrate induced significantly higher MDA levels (Figure 7C). Increases in free proline is also associated with cold tolerance in plants (Ashraf and Foolad, 2007). Increases in proline levels were cold-induced (Figure 7D). Ethylene pathway stimulators resulted in a further increase in free proline content, while ethylene pathway inhibitors had little effect. Interestingly, silver nitrate treatment resulted in a substantial increase in proline levels under control conditions, but not in the cold.

**FIGURE 8:**
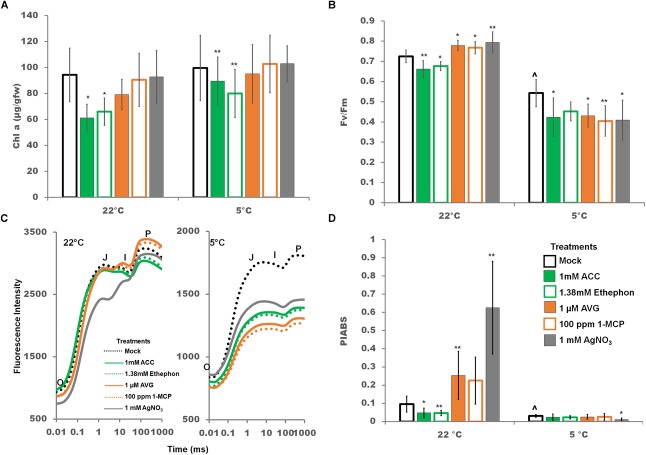
Effects of ethylene pathway manipulation on chlorophyll *a* content and PSII chlorophyll fluorescence parameters. **(A)** Chlorophyll *a* content after 2 days of either control or cold temperatures combined with foliar sprays. **(B)** Quantum yield of photosystem II (F_v_/F_m_) after 2 days of either control or cold temperatures combined with foliar sprays. **(C)** Transient chlorophyll fluorescence (Kautsky curve) plotted on a logarithmic time axis after 2 days of either control or cold temperatures combined with foliar sprays. Averages are represented without error bars for clarity of the graph. **(D)** The Performance Index (PI_ABS_) is a parameter indicating the functionality and capacity of energy capture from PSII through to reduction of PSI. Error bars represent SD. One way ANOVA was performed for each temperature individually (Supplementary Table S3). *Post-hoc* analysis using TukeyHSD was performed comparing treatment with the mock from the same temperature, ^∗^*p* < 0.05, ^∗∗^*p* < 0.01. Comparison between cold treated and non-cold treated mocks was done using Student’s *t*-test, ^∧^*p* < 0.05. *n* = 9.

Manipulation of the ethylene signaling pathway altered PSII photochemistry, and the effect was more pronounced at control temperatures. Chlorophyll *a* content was not affected by a 2 days cold treatment. However, chlorophyll *a* content was reduced at both control and cold temperatures by ethylene pathway stimulators (Figure 8A). A 22°C, ethylene pathway stimulation lowered the quantum photosynthetic efficiency of PSII (F_v_/F_m_), while ethylene pathway inhibition resulted in a significant increase in F_v_/F_m_ indicating that ethylene depresses the efficiency of Q_A_ reduction by captured photons (Figure 8B). In the cold, any manipulation of the ethylene pathway resulted in significantly lower F_v_/F_m_ (Figure 8B). Time resolved transient chlorophyll *a* fluorescent curves (O-J-I-P) can be utilized to dissect the electron flow through the photosystems as each point has been correlated to a physiological state (Strasser and Srivastava, 1995). In dark adapted conditions, all PSII reaction centers are considered open, i.e., capable of accepting excited electrons, at point O, and closed, i.e., completely reduced and incapable of accepting excited electrons, at point P. Briefly O-J relates to the reduction of Q_A_ to ${\text{Q}}_{\text{A}}^{-}$, J-I the reduction of Q_B_ to ${\text{Q}}_{\text{A}}^{2-}$, and I-P the reduction of the PQ pool and electron flow into PSI (Zhu et al., 2005; Boisvert et al., 2006). In the cold, chlorophyll fluorescence transient curves were lower at all states (O, J, I, P) and flattened at the J peak to P peak suggesting disruption in the electron flow from Q_A_ to Q_B_ and beyond (Figure 8C).

At control temperatures, the performance index of photochemistry (PI_ABS_), a structural and functional parameter representing the overall efficiency and capacity of light-dependent photosynthesis, was increased by ethylene pathway inhibition and generally decreased by stimulation (Figure 8D). PI_ABS_ was strongly decreased in the cold. Only silver nitrate treatment resulted in an additional and significant decrease during cold treatment (Figure 8D). These data suggest that ethylene pathway stimulation at control temperatures, cold temperatures, and silver nitrate in the cold all negatively impact overall PSII activity.

Decreases in light dependent photosynthesis functionality and efficiency were noted with ethylene stimulation at control temperatures, cold treatment, and ethylene pathway manipulation in the cold, particularly silver nitrate treatment. Theses decrease are likely being driven by a decrease in PQ pool size limiting electron flow from PSII to PSI as indicated by the shape of the OJIP curve (Figure 8C).

## Discussion

### Soybean Responds Physiologically to Ethylene

The phytohormone ethylene is involved the regulation of many growth and developmental pathways in plants, including senescence and leaf abscission (Grbić and Bleecker, 1995). Accelerated cotyledon abscission was blocked by ethylene pathway inhibitors (silver nitrate and 1-MCP). Likewise the enhanced yellowing of unifoliates caused by ethephon was reduced in presence of silver ions, showing that soybean seedlings respond to ethylene stimulation of senescence and leaf abscission similarly to other plants (Beyer, 1976; Curtis, 1981; Joyce et al., 1990).

### Ethylene and Cold Pathway Crosstalk in Soybean

Ethylene treatment enhances cold tolerance in grapevine (Sun et al., 2016), tomato (Zhang and Huang, 2010), and peanut (Zhang et al., 2016), while in *M. truncatula* (Zhao et al., 2014), Bermuda grass (Hu et al., 2016), and Arabidopsis (Shi et al., 2012) ethylene treatment decreases cold tolerance. The interaction between ethylene and cold in Arabidopsis has been suggested, in the absence of a significant transcriptional response, to be mediated by a cold-stabilized EIN3 protein, which binds to the promoter of *CBF3* preventing its transcription (Shi et al., 2012). In contrast, in soybean, the ethylene signaling pathway is strongly cold-activated, resulting in accumulation of *GmEIN2* and *GmEIN3* transcripts (as well as down regulation of *GmEBF* transcripts), which ultimately results in a decrease in *GmDREB1* transcript levels. Distinctive from the Arabidopsis response (Shi et al., 2012), in soybean this decrease of a key transcription factor in the cold signaling pathway, has little to no impact on cold tolerance in soybean. While silver ions impact both *GmDREB1* transcript levels and freezing tolerance, silver is likely not exerting cold stress enhancement through the ethylene pathway as the more specific ethylene pathway inhibitors, 1-MCP and AVG, have no impact on freezing tolerance despite their strong (and similar to silver) impact on the *GmDREB1* transcripts. As suggested below in the conclusions, silver ions may be enhancing cold tolerance by an alternative mechanism, possibly by induction of an antioxidative response. While the crosstalk between cold and ethylene signaling in soybean shares some similarity with Arabidopsis, the impact on cold tolerance is much different; suggestive that the soybean cold tolerance may not be limited by the CBF/DREB1 cold response.

A putative EIN3 binding motif is present in the promoter of *GmDREB1A:1*, while two EIN3 binding motifs are predicted in *GmDREB1B;1* (Figure 5) suggesting that regulation by GmEIN3s is possible; consistent with the ethylene regulation observed. However, further biochemical studies must be done to demonstrate that GmEIN3A;1 indeed binds to the promoter of *GmDREB1A;1* and *GmDREB1B;1* as predicted. Interestingly, transcripts of *GmDREB1A;2*, which does not possess an obvious EIN3 binding motif, still increased under ethylene inhibition (Figure 5). Closer examination of the *GmDREB1A;2* promoter region revealed a CRT/DRE-like motif (Baker et al., 1994) suggesting that *GmDREB1A;1* could be regulating *GmDREB1A;2*.

### Downstream Targets of GmDREB1s Are Cold Regulated and Ethylene Responsive

All four predicted downstream targets of CBF/DREB1s containing putative CRT/DRE were strongly cold-induced and three were also down-regulated by ethylene pathway stimulation. Only one, ubiquitin ligase, *Glyma.14g212200*, increased following ethylene pathway inhibition (Figure 6). Of the four potential CBF/DREB1 targets, *Glyma.14g212200* was the only transcript with multiple CRT/DRE motifs present in the promoter, which may explain the further enhancement of cold induction with ethylene inhibition (Figure 6). Further studies would be required to determine the mechanism of this regulation.

*GmCYP82A3* (*Glyma.19g014600*), a homolog of the Arabidopsis cytochrome P450 enzyme AtCYP82C (AT4G31940), has been shown to increase in response to iron deficiency and is regulated by the circadian clock (Murgia et al., 2011). In soybean, *GmCYP82A3* transcripts are upregulated by salt and methyl jasmonate, decreased by drought and salicylic acid, and transiently upregulated by ethylene treatment (Yan et al., 2016). In this study, the massive cold induction of *GmCYP823A* is significantly reduced by any ethylene pathway manipulations, both stimulation and inhibition (Figure 6). The promoter of *GmCYP82A3* contains three predicted EIN3 like binding motifs (-148, -284, -1,093 bp) located on the non-coding strand. Each of these EIN3 like binding motifs lack the final 3′ nucleotide found in the consensus AtEIN3 binding motif, ATGYATNY (Konishi and Yanagisawa, 2008; Boutrot et al., 2010) such that these motifs are ATGTATTA, ATGTATGA, ATGTATAG, respectively. The complexity of this response may be explained by *GmCYP82A3* involvement in many abiotic and biotic stress responses and the regulation by these elements.

Two ADH like transcripts were evaluated in this study; only the one with a predicted CRT/DRE present in the promoter (*Glyma.12g015100*) was cold up regulated (Figure 6). In the cold, ethylene pathway activation resulted in a significant decrease for both transcripts; while ethylene pathway inhibition only impacted *Glyma.12g015300*.

### Physiological of Soybean Response to Cold and the Impact of Ethylene Modulators

Soybean is minimally capable of acquiring cold tolerance (Robison et al., 2017). Evidence for increased cold tolerance include decreased electrolyte leakage (Simon, 1974) and increased proline levels (Gilmour et al., 2000). Ethylene pathway inhibition results in an increase in electrolyte leakage in grapevine (Sun et al., 2016) and tomato (Zhang and Huang, 2010) and a decrease in Arabidopsis (Shi et al., 2012), *M. truncatula* (Zhao et al., 2014), and Bermuda grass (Hu et al., 2016). In this study, electrolyte leakage was only significantly improved by treatment with silver nitrate but not other ethylene inhibitors (Figure 7A,B), though both AVG and silver nitrate have been implicated in improving freezing tolerance in the previously mentioned studies. This correlates well with the large singular impact of silver (other ethylene pathway inhibitors had no effect) on the increase in *GmDREB1A;1* transcripts at 22°C and in the massive increase in proline levels. This suggests that the improvement in electrolyte leakage in soybean may have more to do with increases in ROS or other off target effects of silver ion rather than the ethylene signaling pathway.

As MDA is the final product of lipid oxidation (Leshem, 1987), it is often utilized as a proxy for general oxidative damage within plants (Jouve et al., 1993). Ethylene pathway inhibition increases MDA content during heat stress in reproductive soybean (Djanaguiraman et al., 2011). In Bermuda grass, in which ethylene negatively regulates the cold pathway, MDA content increases with ACC treatment and decreases with silver nitrate treatment (Hu et al., 2016). In this study, only 1-MCP resulted in a significant decrease in MDA; with silver nitrate doing the opposite (Figure 7C). In previous studies, transient increases in reactive oxygen species have been suggested to provide abiotic and biotic stress protection (Torres and Dangl, 2005; Suzuki and Mittler, 2006). Low levels of silver causes an increase of super oxide radicals, MDA content and proline levels, as well as increases in superoxide dismutase, peroxidase, and catalase activity; while higher levels decreased antioxidant enzymatic activity and oxygen radicals increased (Qin et al., 2005). The high level of silver used in the present study suggests a significant impact on reactive oxygen species generation.

Proline can play a protective role by stabilizing membranes, buffering redox potential, and as a protein chaperone (Hayat et al., 2012). In this study, proline levels increased slightly with cold. Treatments with 1-MCP and silver nitrate significantly increased proline content at control temperatures, while in the cold ACC and ethephon significantly increased proline content (Figure 7D).

### Photosynthetic Response to Cold and Ethylene

Cold stress can lead to a decrease in the rate and efficiency of photosynthesis (Savitch et al., 2001; Tambussi et al., 2004; Ensminger et al., 2006). Chilling and cold stress effects on soybean photosynthesis have been examined only in late vegetative and reproductive stages (Van Heerden and Kruger, 2000; Van Heerden et al., 2003; Tambussi et al., 2004; Manafi et al., 2015) not in early vegetative stages such as seedlings. In reproductive soybean cold stress (8–9°C) for a 9 h dark period resulted in a decrease in overall light-dependent photosynthesis due to an uneven balance between photon trapping and electron transport from Q_A_ to PSI (Van Heerden and Kruger, 2000; Van Heerden and Krüger, 2002). Data on young seedlings presented here also suggests that light-dependent photosynthesis decreases during extensive cold stress (2 days, 5°C) in seedlings due to an uneven balance between photon trapping and energy transport. This is likely due to a decrease in the PQ pool size in this study leading to less electron transport through the photosystems.

Interestingly, in the cold only silver nitrate treatment significantly impacted PI_ABS_, a structural and functional parameter representing the overall efficiency and capacity of light-dependent photosynthesis. PI_ABS_ was decreased 0.36 fold by silver nitrate treatment indicating that overall silver nitrate is more damaging than any other treatment utilized in this study to light dependent photosynthesis (Figure 8D). As silver nitrate treatment also resulted in a significant increase in MDA content (Figure 7C) it is reasonable to suggest that increased reactive oxygen species are generated by silver nitrate treatment. As photosystem II repair is very sensitive to reactive oxygen species (Nishiyama et al., 2011), we suggest that generation of reactive oxygen species by silver nitrate leads to the further decrease in PI_ABS_ in cold stress soybean seedlings.

Overall, light-dependent photosynthesis in soybean seedlings is negatively impacted by ethylene pathway stimulation at control temperatures, cold temperatures, and ethylene pathway manipulation in the cold through similar mechanisms.

## Conclusion

The response of the *GmEINs* is complex. Soybean *EIN3A;1, EIN3B;1*, and *EIN3C;1* genes were all positively responsive to cold, but only the *EIN3A;1* levels further increased by activation of the ethylene pathway and were repressed by deactivation of the pathway (Figure 4). A downstream target of the EIN3’s, *GmERF056*, was also clearly regulated in the cold, responding as expected in response to alterations in *GmEIN3A;1* transcript levels. The cold responsive transcription factors, *GmDREB1A;1*, and *GmDREB1B;1*, were upregulated by cold and their levels were consistently increased by ethylene pathway inhibitors or decreased (or no effect) by ethylene pathway stimulators (Figure 5). Gene targets with predicted cold responsive elements, CRT/DRE’s, (ADH1A, CYP82A3, Ubiquitin ligase-like, and AP2-like) were indeed cold regulated, while ADH1B which lacks a predicted cold element was not (Figure 6). All these genes responded consistently in the cold by having strongly decreased transcript levels in the presence of stimulators of the ethylene pathway, though interestingly one of these did not have a predicted EIN3 responsive element (AP2-like); suggesting response to an indirect regulator. However, several (AP2-lke, ADH1A, and B) did not respond consistently to ethylene pathway inhibitors; suggesting they might already be at maximal levels in response to cold.

Photosynthetic parameters in soybean were strongly impacted by the cold. This is most dramatically demonstrated by the PI_ABS_ (Figure 8D); the Photosynthetic Performance Index, a measure of the energy conserved from absorption to reduction of PSI acceptors (Figure 8D) (Strasser et al., 2000). The PI_ABS_ is strongly and consistently impacted by both activators and inhibitors of the ethylene pathway, but only under control conditions. Cold conditions appear to bring PI_ABS_ to a minimum level which is little impacted by the ethylene pathway which correlates well with the indicated decreased PQ pool size (Figure 8C).

Not surprisingly, far downstream responses of metabolic indicators of cold stress (Figure 7) were a bit more variable. MDA and proline levels, common responses to cold, were moderately increased in the cold, and only small changes were induced by the presence of ethylene pathway modulators. Interestingly, silver ions alone had a significant impact on both MDA and proline levels, increasing substantially those levels under control conditions. Likewise, silver had a moderate impact on freezing tolerance, decreasing the electrolyte leakage in both acclimated and non-acclimated soybean leaves (Figure 7B). Since these silver effects were not mimicked with either of the ethylene pathway inhibitors AVG or MCP, this suggests off target effects of silver ions, perhaps through its ability to generate free radicals, which can induce oxidative stress and thus oxidative stress responses (Bagherzadeh Homaee and Ehsanpour, 2016; Pradhan et al., 2017). Silver ions have been demonstrated to transcriptionally up-regulate oxidative stress responsive genes such as those encoding superoxide dismutase, cytochrome P450-dependent oxidase, and peroxidase (Kaveh et al., 2013). It may well be that cold tolerance enhancement in soybean by silver is by activating oxidative stress responses as a direct way to moderately increase the cold tolerance of soybean.

Overall, this work has shown that soybean seedlings have a typical ethylene response, indicated by ethylene induced leaf yellowing and cotyledon abscission. Additionally, the ethylene pathway is upregulated in response to cold, mediated by the sustained accumulation of transcripts encoding the transcription factor (*GmEIN3*), the increase in ethylene receptors (*GmETRs*), and the transient loss of transcripts encoding the negative regulatory F-box binding proteins (*GmEBF1s*). Ethylene modulators impact the level of an important cold responding transcription factor family, the *GmCBF/DREB1s*. Not only is the ethylene pathway upregulated in the cold; where it counters the upregulation of cold-responsive genes (*GmCBF/DREB1s*), but at normal temperatures, the cold-responsive genes also appear under negative regulatory control exerted through the ethylene signaling pathway.

**FIGURE 9:**
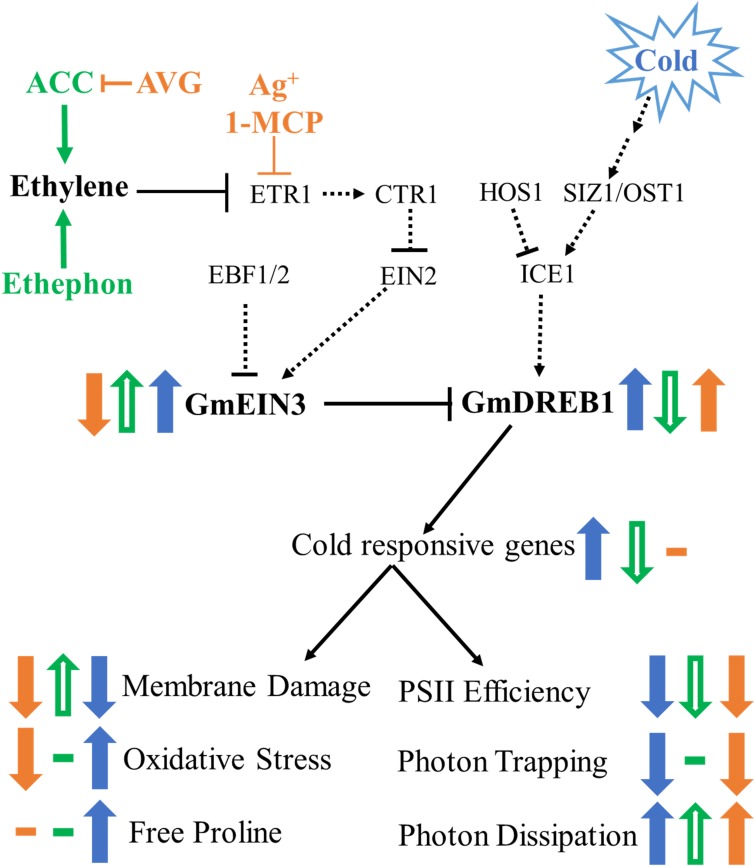
Model of cold stress and ethylene signaling pathway in soybean seedlings. Dashed lines indicated predicted interactions based on Arabidopsis models, solid lines indicate parameters measured in this study. Changes indicated by cold are solid blue arrows, additional changes in the cold due to ethylene pathway stimulation are open green arrows, and ethylene pathway inhibition in solid orange arrows.

Soybean is only mildly cold tolerant and has a meager acclimation response (Robison et al., 2017). Previous work (Yamasaki and Randall, 2016) demonstrated the functionality of the soybean *DREB1* genes by their activation of appropriate targets and resultant enhanced cold tolerance when expressed in Arabidopsis. The observation that up-regulation of the ethylene pathway from the RNA-Seq experiment, in particular the increase in *GmEIN3* transcripts suggested that the low ability of soybean to cold acclimate might be in part due to antagonism by the ethylene pathway. The data described here (summarized in Figure 9) demonstrates a clear impact of the ethylene pathway on the transcription of *GmDREB1s* and their downstream targets; with an activated ethylene pathway leading to decrease of the cold responsive pathway. We suggest that during cold stress GmEIN3A;1 is negatively regulating *GmDREB1A;1* by interaction with the EIN3 binding motif found in the *GmDREB1A;1* promoter. Inhibition of the ethylene pathway resulted in an increase in *GmDREB1A;1* transcript levels. This is quite unlike the impact of altering the ethylene pathway in Arabidopsis (Shi et al., 2012) in that soybean showed no substantial increase in freezing tolerance or cold tolerance parameters. This suggests that the initial portions of the CBF/DREB1 pathway (from cold reception to the accumulation of *GmCBF/DREB1* transcripts) are not limiting the cold responsive pathway in soybean.

## Data Availability

The datasets for the RNAseq experiments can be found on NCBI GEO (Accession # GSE117686) (https://www.ncbi.nlm.nih.gov/geo/query/acc.cgi?acc~=~GSE117686).

## Author Contributions

YY designed and performed the RNASeq analysis and created *AtRD29A_prom_::GFP/GUS* construct for soybean transformation. JR isolated, characterized, and maintained transgenic soybean, designed and completed the ethylene pathway manipulation and cold stress experiments, and composed manuscript with contributions from all authors. SR supervised, designed, analyzed, and conducted experiments and edited the manuscript. All authors reviewed and edited the manuscript.

## Conflict of Interest Statement

The authors declare that the research was conducted in the absence of any commercial or financial relationships that could be construed as a potential conflict of interest.

## Footnotes

1 http://ptf.agron.iastate.edu/
