# Supplementary figures and images for: The Ethylene Signaling Pathway Negatively Impacts CBF/DREB-Regulated Cold Response in Soybean (Glycine max)
Source: Front Plant Sci. 2019 Feb 12;10:121. doi: 10.3389/fpls.2019.00121 (PMC6396728; doi:10.3389/fpls.2019.00121)

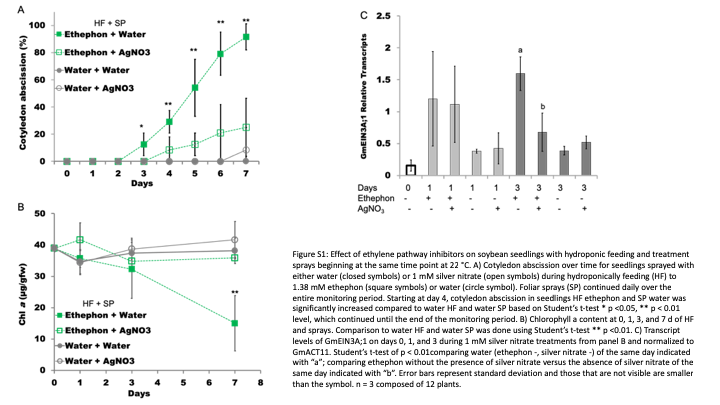

Supplement: Supplementary file 4 [file Image_1.TIFF]

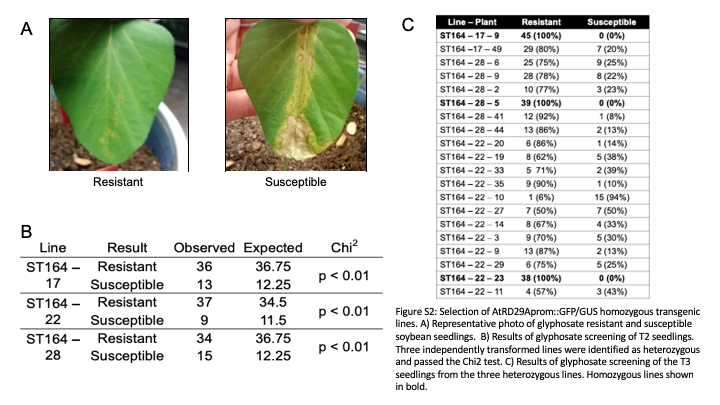

Supplement: Supplementary file 5 [file Image_2.TIFF]

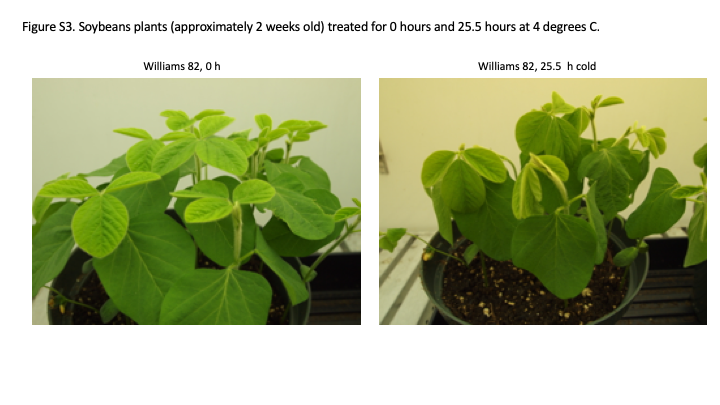

Supplement: Supplementary file 6 [file Image_3.TIFF]
